# A Four-Phenotype Model for Risk Stratification in Heart Failure with Preserved and Mildly Reduced Ejection Fraction: The Role of Sex and Diabetes

**DOI:** 10.3390/biomedicines14010173

**Published:** 2026-01-13

**Authors:** Flavia-Mihaela Stoiculescu, Diana-Ruxandra Hădăreanu, Călin-Dinu Hădăreanu, Maria-Livia Iovănescu, Georgică-Costinel Târtea, Ionuț Donoiu, Petre-Alexandru Cojocaru, Sebastian Militaru, Octavian Istrătoaie, Cristina Florescu

**Affiliations:** 1 Doctoral School, University of Medicine and Pharmacy of Craiova, 200349 Craiova, Romania; 2 Department of Cardiology, Emergency Clinical County Hospital of Craiova, 200642 Craiova, Romania; 3 Department of Cardiology, University of Medicine and Pharmacy of Craiova, 200349 Craiova, Romaniacristina.t.florescu@umfcv.ro (C.F.); 4 Department of Cardiovascular Surgery, Emergency Clinical County Hospital of Craiova, 200642 Craiova, Romania; 5 Department of Cardiology, Filantropia Municipal Hospital Craiova, 200516 Craiova, Romania

**Keywords:** heart failure, heart failure with preserved ejection fraction, heart failure with mildly reduced ejection fraction, sex differences, diabetes mellitus, risk stratification, rehospitalization, prognosis

## Abstract

**Background/Objectives**: Sex and diabetes are important determinants of risk in heart failure with mildly reduced and preserved ejection fraction (HFmrEF/HFpEF), yet their combined effects have not been systematically evaluated. This study examined how sex–diabetes phenotypes influence clinical characteristics and the risk of heart failure rehospitalization. **Methods**: We retrospectively analyzed 1018 HFmrEF/HFpEF patients (2019–2023), classified into four sex–diabetes phenotypes, and performed group comparisons. The primary endpoint was heart failure rehospitalization. **Results**: Over a mean follow-up of 1463 ± 496 days, 307 patients (30.1%) were rehospitalized for heart failure decompensation. The four phenotypes differed significantly in age, renal function, LV mass, LV dimensions, glycemia, and comorbidity burden (all *p* < 0.05). Men—particularly those with diabetes—had greater structural remodeling and higher prevalence of smoking, hypercholesterolemia, and atrial fibrillation. In univariate analysis, male sex, diabetes, smoking, NYHA class, lower TAPSE, and lower LVEF were associated with increased risk of rehospitalization. After adjustment for LVEF and NYHA class, male sex (HR 1.28; *p* = 0.035) and diabetes (HR 1.28; *p* = 0.036) remained independent predictors. Kaplan–Meier curves demonstrated a clear gradient in event-free survival (log-rank *p* = 0.015), with women without diabetes showing the best prognosis and diabetic men the worst. **Conclusions**: Sex and diabetes interact to define distinct risk profiles in HFmrEF/HFpEF. Women without diabetes represent a low-risk phenotype, whereas diabetic men exhibit the highest risk of recurrent heart failure decompensation. These findings support incorporating sex–diabetes phenotyping into routine risk stratification and personalized management.

## 1. Introduction

Heart failure (HF) with mildly reduced (HFmrEF) or preserved ejection fraction (HFpEF) accounts for over half of the global HF burden and continues to rise due to population aging and increasing cardiometabolic disease. Despite its prevalence, HFmrEF/HFpEF remains a heterogeneous syndrome with limited therapeutic options and substantial risk of recurrent decompensation [1]. Identifying factors that modify risk within this population is therefore essential.

Sex strongly shapes HFmrEF/HFpEF pathophysiology and outcomes. Large epidemiologic studies have shown that sex differences influence HF risk across the lifespan, with women experiencing lower lifetime HF mortality but greater symptom burden and a higher prevalence of comorbidities such as obesity, hypertension, and diabetes [2]. Women with HFpEF typically exhibit smaller left ventricular (LV) cavities, concentric remodeling, greater diastolic stiffness, and more severe vascular–ventricular coupling abnormalities [3]. Despite these structural disadvantages, women consistently demonstrate better survival than men in major HFpEF cohorts, despite reporting worse symptoms and functional impairment [4]. In contrast, men present with more ischemic heart disease, greater myocardial fibrosis, higher pulmonary vascular resistance, and worse right ventricular function, factors associated with poorer prognosis [5].

Diabetes mellitus is another major determinant of HF risk and progression. Mechanistically, diabetes promotes microvascular inflammation, endothelial dysfunction, oxidative stress, and myocardial stiffening, key processes implicated in HFmrEF/HFpEF pathophysiology [6]. Epidemiologically, diabetes increases HF risk in both sexes but confers a significantly higher relative excess risk in women, as demonstrated in a meta-analysis of 47 cohorts and over 12 million individuals [7]. Conversely, men with diabetes carry a higher absolute burden of HF events due to greater ischemic and arrhythmic vulnerability [8].

Despite robust evidence that sex and diabetes independently influence HF outcomes, their combined interaction in HFmrEF/HFpEF remains inadequately characterized. Most prior studies have examined sex or diabetes in isolation, without evaluating whether specific sex–diabetes combinations define distinct prognostic endotypes. This gap is clinically relevant, as early mortality is higher in HFmrEF men even when baseline characteristics are matched, whereas HF hospitalization rates often depend more on comorbidity clusters—particularly diabetes—than on sex alone [9].

Therefore, the objective of this study was to investigate the prognostic impact of sex, diabetes, and their interaction on HF rehospitalization in HFmrEF/HFpEF. By identifying whether specific sex–diabetes combinations define distinct clinical risk profiles, this study aims to clarify a currently underexplored source of heterogeneity in HFmrEF/HFpEF and support more individualized approaches to risk stratification.

## 2. Materials and Methods

### 2.1. Study Design and Patient Selection

We conducted a retrospective cohort study using all consecutive discharges from the Clinical Emergency County Hospital of Craiova, Romania, between 1 January 2019 and 31 May 2023. During this interval, the charts from 15,160 consecutive inpatients were analyzed. Individuals were eligible if the index hospitalization resulted in a new diagnosis of HFmrEF or HFpEF, defined according to contemporary guideline criteria [1]. HFmrEF was defined by the presence of HF symptoms and/or signs, a LVEF between 41% and 49%, and additional evidence of cardiac structural or functional abdormalities or elevated natriuretic peptides. HFpEF was defined by the presence of HF symptoms and/or signs, a LVEF above 50%, and evidence of structural or functional abnormalities (such as LV hypertrophy, diastolic dysfunction, left atrial enlargement). Patients were excluded if any of the following conditions were present during the index admission: (1) acute coronary syndrome, (2) acute pulmonary embolism, (3) sustained ventricular tachyarrhythmia or resuscitated cardiac arrest, (4) HF with reduced ejection fraction, (5) second- or third-degree atrioventricular block, (6) missing or incomplete clinical documentation, or (7) insufficient follow-up duration (<1 year) or unavailable follow-up data. The study received approval from the institutional ethics committee (Approval no. 86/19.02.2024). Because of its retrospective nature, the requirement for informed consent was waived, and all patient identifiers were removed before analysis.

### 2.2. Data Collection and Assessment

Baseline demographic, clinical, and therapeutic information was retrieved from electronic medical records, including age, sex, heart rhythm, vital signs, New York Heart Association (NYHA) functional class at discharge, cardiovascular risk factors, medication use, and prior medical history.

Laboratory parameters collected at discharge included hemoglobin, hepatic enzymes, serum creatinine, serum sodium and potassium, and NT-proBNP. Estimated glomerular filtration rate (eGFR) was calculated using the CKD-EPI equation.

Comprehensive echocardiographic evaluation was performed in accordance with the recommendations of the European Association of Cardiovascular Imaging [10]. Measurements included left and right ventricular and atrial dimensions, LVEF using the Simpson biplane method, E/A ratio, tricuspid annular plane systolic excursion (TAPSE), tricuspid regurgitation peak velocity, and systolic pulmonary artery pressure, derived from the tricuspid regurgitation jet and inferior vena cava indices. LV mass was calculated with the Devereux formula, and relative wall thickness was obtained as: 2 × posterior wall thickness/LV end-diastolic diameter.

As echocardiographic data was obtained from archive clinical reports, in which left atrial volume index was not consistently available, to enusre internal validity and minimize missing data, left atrial diameter was used as a surrogate.

Echocardiographic examinations in our study were performed as part of routine clinical care by board-certified cardiologists with echocardiography accreditation according to national and European training standards. However, because of the retrospective design and reliance on existing clinical records, formal intra- and inter-observer variability assessments were not available.

Conventional risk factors were recorded as follows: smoking status (current or former), dyslipidemia (LDL-cholesterol > 130 mg/dL or lipid-lowering treatment), hypertension (BP ≥ 140/90 mmHg or antihypertensive therapy), type 2 diabetes mellitus, and chronic kidney disease.

Ischemic heart disease was determined based on clinical evaluation and available coronary imaging. Most patients with suspected coronary disease underwent invasive coronary angiography; coronary CT angiography was rarely used. Ischemic etiology was assigned when at least one of the following was documented: significant obstructive stenosis (>70% in a major epicardial artery or >50% in the left main), prior myocardial infarction, or prior revascularization. Patients with previous percutaneous intervention but no infarction were also considered to have ischemic HF. In patients without angiography and low clinical suspicion, ischemia may have remained unconfirmed. Valvular heart disease was defined as moderate or severe stenosis or regurgitation. Congenital heart disease encompassed any congenital structural cardiac abnormality recorded in the medical history (e.g., atrial or ventricular septal defects, bicuspid aortic valve, Ebstein anomaly, atrioventricular canal defects, or repaired tetralogy of Fallot).

### 2.3. Follow-Up and Study Endpoint

The primary endpoint was first rehospitalization for heart failure decompensation. Rehospitalization events were identified based on hospital discharge diagnoses recorded in the institutional electronic medical records. For patients followed outside the index hospital, follow-up information was supplemented by structured outpatient visits and telephone contact when available. Independent adjudication of events was not performed due to the retrospective study design. Mortality status was verified using each individual’s national identification number. The chosen endpoint was first rehospitalization for HF decompensation, defined as an unplanned hospital admission requiring intravenous diuretics, vasodilators, or inotropes for HF worsening. For patients without events, follow-up was censored at the date of last contact. For those with multiple HF readmissions, only the first event was used in time-to-event analyses.

### 2.4. Statistical Analysis

The normality of continuous variables was assessed using the Shapiro–Wilk test. Continuous data are reported as means ± standard deviations (SDs), while categorical variables are expressed as counts and percentages.

Variables were compared across the four sex–diabetes groups. For normally distributed continuous variables, one-way ANOVA was applied together with Levene’s test for homogeneity of variances; when variances were equal, post hoc pairwise comparisons were performed with Tukey’s test, and when variances were unequal, the Games–Howell procedure was used. Categorical variables were compared using the Pearson chi-square test. For each contingency table, SPSS-generated adjusted standardized residuals were examined to identify which cells contributed to statistically significant differences. Residuals with an absolute value ≥ 1.96 were considered significant at *p* < 0.05, ≥2.58 at *p* < 0.01, and ≥3.29 at *p* < 0.001. Positive residuals indicated that the observed frequency was higher than expected under the null hypothesis, while negative residuals indicated a lower-than-expected frequency. This method allowed post hoc identification of pairwise group differences without conducting multiple chi-square tests. Variables with missing observations were analyzed according to the number of valid cases reported by SPSS.

Univariable Cox proportional hazards models were used to explore associations between baseline clinical, biochemical, and echocardiographic parameters and the study endpoints. Variables included in the multivariable Cox model were selected a priori based on clinical relevance rather than on univariate statistical significance. This approach avoids confounder omission and ensures appropriate adjustment when evaluating the independent prognostic contribution of sex and diabetes.

Kaplan–Meier curves were generated to assess event-free survival, and differences were evaluated using the log-rank test.

A two-sided *p* value < 0.05 was considered statistically significant. Analyses were performed using available-case data for each variable, as the extent of missingness varied across parameters. All analyses were conducted using SPSS (version 23).

## 3. Results

### 3.1. Study Population and Baseline Characteristics

A total of 1018 patients represented the final study population. The mean follow-up duration was 1463 ± 496 days (approximately 4 years). During follow-up, 307 patients (30.1%) met the primary endpoint of rehospitalization for heart failure decompensation.

The cohort had a mean age of 74 ± 11 years, and comorbidity burden was high: hypertension (79%), hypercholesterolemia (65%), atrial fibrillation (48%), type 2 diabetes mellitus (36.7%), chronic kidney disease (34%), and a history of smoking (14%). Most patients were in NYHA class II–III, with a mean LVEF of 49 ± 6%.

Across the four sex–diabetes phenotypes, several significant differences were found between baseline characteristics (Table 1). Women without diabetes were the oldest group, whereas diabetic men were the youngest (*p* < 0.001). Diabetic patients—both men and women—had significantly higher blood glucose levels compared with their non-diabetic counterparts (*p* < 0.001). Men (with and without diabetes) exhibited larger LV dimensions and greater LV mass than women, with all pairwise comparisons showing statistically significant differences (*p* < 0.001). Women without diabetes had higher eGFR values compared with both male groups and diabetic women (*p* = 0.001). Finally, diabetic men had the shortest time to first rehospitalization compared with women without diabetes and non-diabetic men (*p* = 0.012).

Smoking was markedly more prevalent among non-diabetic and diabetic men, while women—especially those without diabetes—had lower smoking rates. The prevalence of hypercholesterolemia varied substantially, with diabetic men showing the highest rates. Atrial fibrillation was significantly more common among diabetic men, whereas non-diabetic women had lower-than-expected atrial fibrillation prevalence. Fatigue was more frequently reported by diabetic patients, particularly men. Conversely, chest pain and edema showed no significant distribution differences between groups. Severity grades of mitral and tricuspid regurgitation also displayed heterogeneous distributions, driven primarily by higher grades observed in male patients.

Use of beta-blockers, ACE inhibitors/angiotensin receptor–neprilysin inhibitors, mineralocorticoid receptor antagonists, loop diuretics, and amiodarone did not differ significantly across phenotypes. In contrast, SGLT2 inhibitor use differed significantly, being most frequent among diabetic men.

### 3.2. Cox Regression Analysis

In univariate Cox models (Table 2) evaluating the risk of HF rehospitalization, male sex (HR = 1.324, 95% CI 1.055–1.662, *p* = 0.015) and type 2 diabetes mellitus (HR = 1.304, 95% CI 1.038–1.639, *p* = 0.023) were both associated with a higher risk of rehospitalization. Active smoking also increased risk (HR = 1.206, 95% CI 1.016–1.432, *p* = 0.032). Worse functional status reflected by a higher NYHA class strongly predicted adverse outcomes (HR = 1.289, 95% CI 1.081–1.537, *p* = 0.005). Among echocardiographic measures, reduced TAPSE was a significant predictor of rehospitalization (HR = 0.937, 95% CI 0.894–0.982, *p* = 0.007), and higher LVEF was modestly protective (HR = 0.982, 95% CI 0.964–0.999, *p* = 0.039), consistent with the pathophysiological spectrum of HFmrEF/HFpEF.

Variables entered into the multivariate Cox proportional hazards model were selected based on clinical relevance rather than univariate statistical significance, in order to avoid omitted-variable bias. The final model included male sex, diabetes status, NYHA class, and LVEF.

After adjustment, male sex (HR 1.28, 95% CI 1.02–1.61, *p* = 0.035) and diabetes (HR 1.28, 95% CI 1.02–1.61, *p* = 0.036) remained independently associated with a higher risk of the composite outcome. NYHA class was also independently associated with the outcome (HR 1.24 per class increase, 95% CI 1.04–1.49, *p* = 0.018). LVEF did not remain independently significant in the adjusted model (HR 0.99, *p* = 0.234).

To evaluate whether sex–diabetes phenotypes predicted HF rehospitalization beyond comorbidity burden, an additional Cox regression model was constructed including age, estimated glomerular filtration rate, and atrial fibrillation. After adjustment, the overall association between phenotype and outcome was attenuated (*p* = 0.054). Importantly, diabetic men continued to exhibit a significantly higher risk of HF rehospitalization compared with non-diabetic women (HR 1.58, 95% CI 1.14–2.18; *p* = 0.006), whereas other phenotypes were not independently associated with outcome.

### 3.3. Kaplan–Meier Survival Analysis

**Figure 1:**
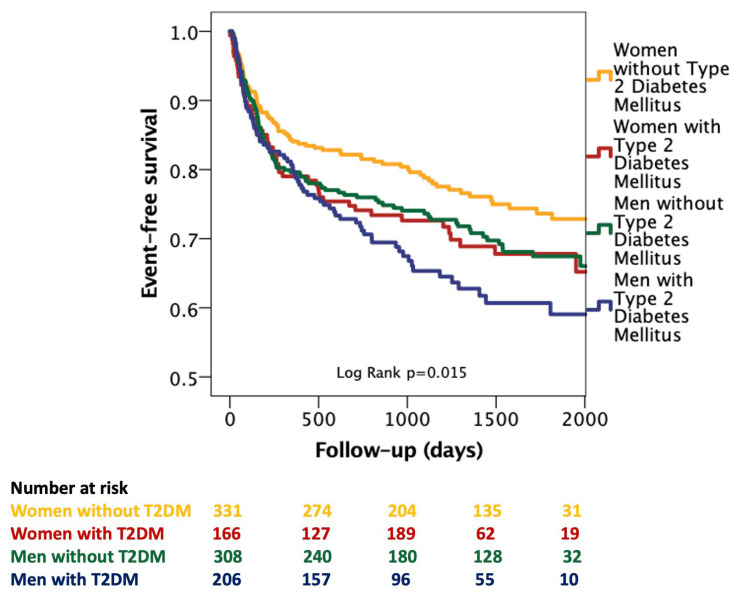
Kaplan–Meier curves showing event-free survival across the four sex–diabetes phenotypes. Women without diabetes had the most favorable survival, whereas diabetic men showed the steepest early decline and worst long-term prognosis (log-rank *p* = 0.015). Abbreviations: T2DM, type 2 Diabetes Mellitus.

Kaplan–Meier survival curves (Figure 1) demonstrated significant differences in event-free survival across the four sex–diabetes groups (log-rank *p* = 0.015). Women without diabetes showed the most favorable prognosis, maintaining the highest survival probability throughout follow-up. Women with diabetes and men without diabetes displayed intermediate survival trajectories with comparable event-free survival curves. In contrast, men with diabetes experienced the poorest outcomes, exhibiting the steepest early decline and the lowest long-term event-free survival. This pattern highlights a clear interaction between sex and diabetic status, with the combination of male sex and diabetes conferring the greatest risk of adverse events.

## 4. Discussion

In this study of 1018 patients with HFmrEF/HFpEF, we investigated how sex and diabetes jointly influence clinical profiles and long-term outcomes. The main findings were that women without diabetes exhibited the most favorable prognosis, diabetic women and non-diabetic men showed intermediate risk, and diabetic men consistently demonstrated the highest likelihood of HF rehospitalization. This stepwise gradient appeared across baseline characteristics, structural and functional parameters, survival curves, and multivariable models. Importantly, our work provides novel insight by directly comparing combined sex–diabetes phenotypes within a single HFmrEF/HFpEF cohort—an approach not systematically explored in previous studies, which typically examined sex or diabetes effects in isolation. By evaluating these modifiers together, we highlight a biologically coherent and clinically meaningful interaction that helps explain the heterogeneous outcomes observed in patients with preserved-range EF.

### 4.1. Sex-Specific Differences in HFmrEF/HFpEF

Previous epidemiologic and imaging studies have shown that women with HFpEF generally exhibit smaller LV cavities, greater concentric remodeling, higher ventricular–arterial elastance, and lower myocardial fibrosis [3]. These structural advantages correlate with the consistently lower mortality observed among women in trials such as CHARM-Preserved, I-Preserve, and TOPCAT [4]. In line with these findings, women without diabetes in our cohort showed the most favorable survival, suggesting that preserved diastolic reserve, superior microvascular function, and lower fibrosis burden contribute to a protective HF phenotype.

Men, on the other hand, demonstrated a phenotype characterized by larger LV volumes, increased fibrosis, greater arrhythmia burden, and worse right ventricular–pulmonary artery coupling [3,5]. These pathophysiological features likely underlie the poorer outcomes observed in both diabetic and non-diabetic men in our study. Notably, even non-diabetic men had worse prognosis than diabetic women, consistent with registry data showing male sex as an independent predictor of cardiac mortality [5].

### 4.2. Impact of Diabetes and Sex-Diabetes Interaction

Diabetes emerged as a key modifier of HF risk. Previous studies, including DIAMOND-CHF [11] and a meta-analysis [7], demonstrated a disproportionately higher relative HF risk in diabetic women. Mechanisms include enhanced endothelial dysfunction, microvascular rarefaction, inflammation, and loss of estrogen-mediated vascular protection [3,6]. In our cohort, diabetic women had significantly worse outcomes than non-diabetic women, and categorical findings—such as increased fatigue, atrial fibrillation prevalence, and valvular dysfunction—suggested that diabetes may accelerate structural deterioration in women.

Further support for the strong influence of diabetes on HF progression comes from metabolic and epidemiological studies [12]. Diabetes induces a shift from glucose oxidation toward fatty-acid oxidation, leading to impaired cardiac energetics, reduced contractile efficiency, and both systolic and diastolic dysfunction, even in the absence of coronary disease [13]. Hyperglycemia and insulin resistance additionally promote microvascular dysfunction, increased oxidative stress, local activation of the renin–angiotensin and sympathetic nervous systems, and enhanced myocardial fibrosis [13]. These metabolic disturbances explain why epidemiological data consistently demonstrate that diabetes increases the risk of developing HF more than twofold in men and more than fivefold in women [14,15], reinforcing the sex-specific vulnerability described in our cohort. Moreover, diabetes substantially increases recurrent HF hospitalizations and mortality across both reduced and preserved EF phenotypes, aligning with the markedly higher rehospitalization rates observed among diabetic patients in our study. The CHARM program similarly reported a twofold higher risk of cardiovascular death or HF hospitalization in insulin-treated diabetics [15]. Collectively, these data strengthen the biological plausibility of our findings, supporting diabetes as a major driver of adverse remodeling and HF morbidity, and highlighting the importance of integrating metabolic status into risk stratification frameworks.

However, despite this higher relative risk in women, men consistently experience a greater absolute burden of adverse HF events [8,16]. This pattern was reflected in our cohort: diabetic men had the highest comorbidity burden, most adverse remodeling, and the steepest decline in event-free survival. Thus, although diabetes confers a stronger relative risk in women, its absolute effect is most severe in men. These findings suggest that sex–diabetes phenotypes capture both biological differences and clustering of adverse comorbidities, with diabetic men representing a particularly high-risk subgroup even after adjustment for age, renal function, and atrial fibrillation. The observed association between atrial fibrillation and lower rehospitalization risk likely reflects differences in monitoring intensity and healthcare contact rather than a protective biological effect.

Although part of the observed risk gradient may reflect clustering of comorbidities across the four phenotypes, major HFpEF and HFmrEF trials and registries have consistently demonstrated that sex and diabetes are not merely correlates of comorbidity burden, but key modifiers of phenotype and prognosis. Sex-related differences in outcomes and clinical profiles have been reported across trials such as CHARM-Preserved [15], I-Preserve [17], and TOPCAT [18], with women demonstrating better survival despite a substantial comorbidity burden. Furthermore, diabetes has repeatedly been associated with higher rates of adverse outcomes in heart failure, including in the CHARM study. In this context, our findings are unlikely to be explained solely by comorbidity clustering and instead appear to reflect distinct risk phenotypes in which sex and diabetes act as meaningful modifiers within a broader comorbidity framework.

### 4.3. Structural, Functional, and Comorbidity Differences Across Phenotypes

Sex-specific anatomical and physiological differences further shape HF outcomes. Women generally have smaller LV size, lower stroke volume, and higher pulsatile load [19,20], while men more frequently develop ischemic scar and eccentric remodeling [21]. With diabetes, these differences intensify: women exhibit more pronounced concentric LV hypertrophy, while men demonstrate greater fibrosis and ischemic injury [21]. Additional evidence shows faster HF progression and more pronounced LV hypertrophy in diabetic patients, even at lower body-mass index, driven by oxidative stress, metabolic dysfunction, microvascular impairment, and extracellular matrix remodeling [22].

In our study, baseline continuous and categorical variables reinforced this gradient. Women without diabetes were the oldest yet had the most preserved renal function. Men—especially diabetic men—showed larger LV dimensions, greater LV mass, lower TAPSE, more atrial fibrillation, higher smoking prevalence, and more severe tricuspid regurgitation and mitral regurgitation grades. Diabetic patients exhibited higher glucose levels and shorter time to first rehospitalization, emphasizing the close link between metabolic dysfunction and HF progression.

### 4.4. Prognostic and Clinical Implications

Univariate Cox regression identified male sex, diabetes, smoking, NYHA class, TAPSE, and LVEF as significant predictors of rehospitalization. After adjustment for NYHA class and LVEF, male sex and diabetes remained independent predictors, underscoring their strong and consistent prognostic effect across HFmrEF/HFpEF. Kaplan–Meier curves showed a clear, stepwise separation of the four phenotypes, with women without diabetes showing the best outcomes and diabetic men the worst.

Taken together, these findings support a unified mechanistic framework. Women without diabetes appear to maintain a distinctly favorable cardiovascular profile, characterized by preserved microvascular function, less fibrosis, and more efficient ventricular–vascular coupling. The presence of diabetes alters this otherwise advantageous profile in women, shifting them into an intermediate-risk category by amplifying metabolic stress, microvascular dysfunction, and diastolic impairment. Men, even in the absence of diabetes, display a phenotype that is intrinsically more vulnerable, marked by greater myocardial fibrosis, more pronounced remodeling, and higher arrhythmic and ischemic burden. When diabetes is added to this already high-risk male substrate, the combined effect results in the most pronounced structural deterioration and the poorest clinical outcomes, producing the highest likelihood of recurrent HF decompensation observed in our cohort. This model aligns with the mechanistic and epidemiologic patterns previously described [3,8,16].

Clinically, our findings reinforce the need for sex-specific and diabetes-specific risk stratification in HFmrEF/HFpEF. Diabetic women require aggressive metabolic and cardiovascular risk control given their disproportionate relative risk, whereas diabetic men represent a uniquely high-risk endotype requiring intensified ischemic, arrhythmic, and RV-focused evaluation. Recognizing non-diabetic women as the lowest-risk group may refine follow-up intensity and therapeutic strategies. Incorporating sex–diabetes phenotyping into routine clinical assessment may therefore enhance personalized prognostication and improve management across the HFmrEF/HFpEF spectrum.

### 4.5. Limitations

This study has several limitations. First, its retrospective, single-center design may limit generalizability and introduces the risk of residual confounding. Second, stratifying patients into four sex–diabetes phenotypes, although essential to the study’s aim, reduced statistical power for some subgroup comparisons. Third, key variables such as glycemic control, diabetes duration, HbA1c values, diabetes-related complications, detailed antidiabetic treatment and detailed ischemic burden were not consistently available and may have influenced outcomes. Medication use was assessed at discharge only, without information on dose, duration, or adherence. The higher prevalence of SGLT2 inhibitor use among diabetic men likely reflects confounding by indication rather than treatment effect. In addition, anthropometric measurements such as body mass index (BMI) and body surface area (BSA) were not included because height was not systematically recorded, precluding accurate calculation for most patients; to avoid bias arising from incomplete or inconsistent data, BMI-, BSA-, and related indexed variables were therefore not analyzed. Consequently, the inability to account for glycemic control, diabetes severity and intensity of treatment may have led to residual confounding in the observed associations. Fourth, echocardiographic measurements were obtained from routine clinical practice rather than core-lab adjudication, allowing for potential measurement variability. Advanced echocardiographic parameters with demonstrated prognostic value in patients with HF were not included due to the retrospective design of the study, and the use of left atrial diameter instead of indexed left atrial volume may have underestimated atrial remodeling compared with guideline-recommended parameters. Finally, heart failure rehospitalization—while clinically relevant—may be affected by healthcare access and admission thresholds, and, given the retrospective design and reliance on discharge diagnoses, misclassification of events cannot be excluded, particularly for admissions occurring outside the study center.

## 5. Conclusions

Sex and diabetes strongly influence the risk of HF rehospitalization in HFmrEF/HFpEF, with women without diabetes experiencing the lowest risk and diabetic men the highest. Diabetes appears to attenuate the protective advantage seen in women, while male sex is associated with greater diabetes-related vulnerability, potentially reflecting underlying risk clustering. Our findings support integrating sex–diabetes profiling into routine risk assessment and management.

## Figures and Tables

**Table 1:** Baseline characteristics across the four sex-diabetes phenotypes.

Variable	All Patients (N = 1018)	Non-Diabetic Women (G1, N = 334)	Diabetic Women (G2, N = 167)	Non-Diabetic Men (G3, N = 310)	Diabetic Men (G4, N = 207)	ANOVA *p*-Value
Age (years)	74 ± 11	76 ± 11	75 ± 9	73 ± 13	71 ± 10	<0.001 ^1^
Heart rate (bpm)	73 ± 14	73 ± 12	75 ± 15	71 ± 14	73 ± 14	0.031 ^2^
Systolic blood pressure (mmHg)	130 ± 20	129 ± 20	132 ± 20	128 ± 21	132 ± 19	0.064
Diastolic blood pressure (mmHg)	76 ± 11	75 ± 11	78 ± 11	74 ± 11	76 ± 11	0.011 ^3^
Time to first rehospitalization (days)	1133 ± 683	1196 ± 667	1114 ± 697	1161 ± 707	1004 ± 646	0.012 ^4^
NT-proBNP (pg/mL)	5547 ± 7544	6109 ± 7213	6470 ± 8554	5275 ± 8313	4132 ± 5555	0.165
Creatinine (mg/dL)	1.2 ± 0.7	1.1 ± 0.6	1.2 ± 0.6	1.3 ± 0.9	1.3 ± 0.6	0.001 ^5^
eGFR (mL/min/1.73 m^2^)	73 ± 25	77 ± 24	73 ± 25	71 ± 27	70 ± 24	0.001 ^6^
Glucose (mg/dL)	123 ± 55	110 ± 42	148 ± 66	107 ± 25	148 ± 74	<0.001 ^7^
Serum sodium (mmol/L)	138 ± 6	138 ± 6	139 ± 9	138 ± 4	138 ± 4	0.470
Interventricular septum width (mm)	13 ± 2	12 ± 2	13 ± 2	13 ± 3	13 ± 2	0.001 ^8^
Left ventricular end-diastolic diameter (mm)	50 ± 7	47 ± 6	48 ± 6	52 ± 8	52 ± 7	<0.001 ^9^
Posterior wall width (mm)	12 ± 2	12 ± 2	12 ± 2	12 ± 2	13 ± 2	0.034 ^10^
Left ventricular mass (g)	241 ± 78	216 ± 67	230 ± 64	257 ± 85	268 ± 80	<0.001 ^11^
Relative wall thickness	0.49 ± 0.13	0.50 ± 0.14	0.52 ± 0.14	0.47 ± 0.13	0.48 ± 0.11	<0.001 ^12^
Left atrial diameter (mm)	48 ± 10	48 ± 11	47 ± 7	48 ± 11	47 ± 8	0.133
E/E′	14 ± 3	12 ± 3	13 ± 2	14 ± 3	14 ± 4	0.856
Systolic pulmonary artery pressure (mmHg)	51 ± 17	51 ± 16	55 ± 20	48 ± 17	50 ± 18	0.078
Tricuspid annulus plane systolic excursion (mm)	19 ± 5	19 ± 5	19 ± 6	18 ± 4	19 ± 4	0.949
Left ventricular ejection fraction (%)	49 ± 6	51 ± 7	50 ± 6	49 ± 6	48 ± 6	0.001 ^13^

Superscript numbers correspond to significant post hoc pairwise comparisons listed below the table: ^1^ Age: G1 > G3 (*p* = 0.005); G1 > G4 (*p* < 0.001); G2 > G4 (*p* = 0.001). ^2^ Heart rate: G2 > G3 (*p* = 0.020). ^3^ Diastolic blood pressure: G2 > G3 (*p* = 0.009). ^4^ Time to rehospitalization: G1 > G4 (*p* = 0.006); G3 > G4 (*p* = 0.046). ^5^ Creatinine: G1 < G3 (*p* = 0.001); G1 < G4 (*p* = 0.024). ^6^ eGFR: G1 > G3 (*p* = 0.005); G1 > G4 (*p* = 0.004). ^7^ Glucose: G1 < G2 (*p* < 0.001); G1 < G4 (*p* < 0.001); G2 > G3 (*p* < 0.001); G3 < G4 (*p* < 0.001). ^8^ Interventricular septum width: G1 < G2 (*p* = 0.031); G1 < G4 (*p* = 0.001). ^9^ Left ventricular end-diastolic diameter: G1 < G3 (*p* < 0.001); G1 < G4 (*p* < 0.001); G2 < G3 (*p* < 0.001); G2 < G4 (*p* < 0.001); G3 < G4 (*p* < 0.001). ^10^ Posterior wall width: G1 < G4 (*p* = 0.028); ^11^ Left ventricular mass: G1 < G3 (*p* < 0.001); G1 < G4 (*p* < 0.001); G2 < G3 (*p* = 0.001); G2 < G4 (*p* < 0.001). ^12^ Relative wall thickness: G1 > G3 (*p* = 0.009); G2 > G3 (*p* = 0.001); G2 > G4 (*p* = 0.017). ^13^ Left ventricular ejection fraction: G1 > G3 (*p* = 0.040); G1 > G4 (*p* = 0.011); G2 > G3 (*p* = 0.032); G2 > G4 (*p* = 0.010); G3 > G4 (*p* = 0.032).

**Table 2:** Univariate Cox regression analysis.

Variable	*p*-Value	HR (95% CI)
Age	<0.001	0.983 [0.974–0.992]
Sex (male)	0.015	1.324 [1.055–1.662]
Type 2 diabetes mellitus	0.023	1.304 [1.038–1.639]
Hypertension	0.011	0.714 [0.552–0.925]
Hypercholesterolemia	0.949	1.008 [0.795–1.277]
Smoking	0.032	1.206 [1.016–1.432]
Chronic kidney disease	0.759	1.038 [0.820–1.314]
NYHA class	0.005	1.289 [1.081–1.537]
Sodium-glucose cotransporter-2 inhibitors use	0.002	1.780 [1.233–2.571]
Betablockers use	0.526	1.107 [0.809-1.515]
Loop diuretics use	0.078	1.333 [0.969–1.835]
Left ventricular mass	0.156	1.001 [1.000–1.002]
Relative wall thickness	0.480	0.734 [0.311–1.733]
Systolic pulmonary artery pressure	0.311	1.005 [0.995–1.016]
Tricuspid regurgitation severity grade	0.681	1.021 [0.923–1.130]
Mitral regurgitation severity grade	0.186	1.083 [0.962–1.218]
Tricuspid annulus plane systolic excursion	0.007	0.937 [ 0.894–0.982]
Left ventricular ejection fraction	0.039	0.982 [0.964–0.999]

Abbreviations: CI, confidence interval; HR, hazard ratio.

## Data Availability

The raw data supporting the conclusions of this article will be made available by the authors upon reasonable request and following approval by the University of Medicine and Pharmacy of Craiova, Romania. The data presented in this study are not publicly available due to privacy and ethical restrictions.

## Abbreviations

The following abbreviations are used in this manuscript:

BMI	Body mass index
BSA	Body surface area
EF	Ejection fraction
eGFR	Estimated glomerular filtration rate
ESC	European Society of Cardiology
HF	Heart failure
HFmrEF	Heart failure with mildly reduce ejection fraction
HFpEF	Heart failure with preserved ejection fraction
HR	Hazard ratio
LV	Left ventricle/ventricular
LVEF	Left ventricular ejection fraction
NT-proBNP	N-terminal pro-B-type natriuretic peptide
NYHA	New York Heart Association
TAPSE	Tricuspid annular plane systolic excursion
